# Single-cell and bulk transcriptome analyses reveal elevated amino acid metabolism promoting tumor-directed immune evasion in colorectal cancer

**DOI:** 10.3389/fimmu.2025.1575829

**Published:** 2025-05-22

**Authors:** Tianyue Sun, Yan Chen, Ying-Xuan Chen

**Affiliations:** ^1^ State Key Laboratory for Oncogenes and Related Genes, Division of Gastroenterology and Hepatology, Renji Hospital, School of Medicine, Shanghai Jiao Tong University, Shanghai, China; ^2^ Department of Gastroenterology, Sichuan Provincial People’s Hospital, School of Medicine, University of Electronic Science and Technology of China, Chengdu, China

**Keywords:** colorectal cancer, amino acid metabolism, tumor metabolic reprogramming, tumor immune evasion, immunotherapy, single cell RNA and transcriptome sequencing

## Abstract

**Introduction:**

Colorectal cancer (CRC), the third most common cancer worldwide, often shows limited responsiveness to immunotherapy due to its predominantly immune-excluded phenotype. Despite increasing insights into the complex tumor microenvironment (TME), the metabolic heterogeneity of CRC cells and their interactions with tumor-infiltrating immune cells remain poorly understood.

**Methods:**

We analyzed 46,374 epithelial cells from 17 CRC patients treated with PD-1 blockade to develop an amino acid (AA) metabolism score using the AUCell algorithm. This score was applied to a separate single-cell RNA sequencing (scRNA-seq) dataset from 23 CRC patients to investigate cell-cell interactions and functions of tumor-infiltrating immune cells, revealing distinct immune TME landscapes shaped by tumor metabolism. An *in vitro* co-culture assay of CRC cells and CD8^+^ T cells was performed to validate the findings. Additionally, LASSO and Cox regression analyses were conducted to construct an AA metabolism-related risk score for predicting prognosis and drug sensitivity across multiple bulk transcriptome cohorts.

**Results:**

This study identified a link between elevated amino acid metabolism in CRC epithelial cells and resistance to PD-1 blockade therapy. A 31-gene AA score was developed by intersecting differentially expressed genes between responders and non-responders to PD-1 blockade with amino acid metabolism-related genes from the Molecular Signature Database (MSigDB). Using this score, 23 additional CRC samples were classified into high and low AA score groups. Comparative analysis revealed that the low AA group exhibited a more robust immune response, characterized by a greater number and stronger cell-cell interactions. Tumor-infiltrating immune cells in this group demonstrated enhanced activation and anti-tumor functions. Furthermore, CD8^+^ T cells showed increased Granzyme B levels when co-cultured with CRC cells in which Psat1 or Shmt2 was knocked down. Finally, a machine learning-derived risk score based on six genes was established to translate single-cell findings to bulk transcriptomes. This risk score was found to correlate with immune checkpoint expression and immune cell infiltration, with potential implications for predicting prognosis and drug sensitivity.

**Conclusion:**

Our findings highlight the role of elevated epithelial amino acid metabolism in shaping an immune-suppressive microenvironment, offering insights for patient stratification and therapeutic decision-making.

## 1 Introduction

Cancer immunotherapies have revolutionized oncological treatment, with immune checkpoint blockade (ICB) therapies, such as PD-1 inhibitors, achieving significant success (1). However, the efficacy of these therapies varies among cancer patients and types (2). Colorectal cancer (CRC), often described as a ‘cold’ tumor for its predominantly immune-excluded phenotype (3), remains the second leading cause of cancer-related deaths worldwide (4). CRC patients with defective mismatch repair or microsatellite instability-high (dMMR/MSI-H) tumors generally respond better to PD-1 blockade due to a higher tumor neoantigen load and increased immune cell infiltration (5, 6). In contrast, the majority of CRC patients with mismatch repair-proficient and microsatellite-stable (pMMR/MSS) tumors show limited or no response to ICB therapy, presenting a major challenge to clinical management and patient survival.

Metabolic reprogramming is a well-established hallmark of cancer (7). Advances in understanding of the TME reveal that tumor metabolism not only fuels uncontrolled proliferation but also facilitates immune evasion (8). Tumor cells compete with immune cells for nutrients and release specific metabolites into the TME, which impair the anti-tumor functions of tumor-infiltrating immune cells (9–11). However, the metabolic heterogeneity of colorectal epithelium and its interactions with immune cells remain poorly understood. Nonetheless, scRNA-seq provides an unprecedented opportunity to investigate key metabolic pathways, cellular diversity, and phenotypic heterogeneity of various cell types at single-cell resolution.

In this study, we explored the role of amino acid metabolism in CRC epithelial cells, revealing its association with response to PD-1 blockade therapy through scRNA-seq. Our analysis identified distinct immune TME landscapes shaped by tumor amino acid metabolic reprogramming. We also applied these findings to bulk transcriptome data, developing an amino acid metabolism-based risk score that correlates with immune cell infiltration, immune checkpoint expression, and patient survival. Our study revealed how tumor cell amino acid metabolism reprogramming promoted immune evasion by altering the tumor microenvironment, with potential implications for risk stratification and personalized treatment strategies in CRC.

## 2 Materials and methods

### 2.1 Data acquisition

The scRNA-seq datasets were obtained from GSE205506 (12) and GSE132465 (13) in the Gene Expression Omnibus (GEO) database (https://www.ncbi.nlm.nih.gov/gds). Bulk mRNA arrays were also downloaded from GSE39582 (14) in GEO database. TCGA-COAD bulk RNA sequencing (RNA-seq) data and survival information were downloaded through UCSC Xena browser (https://xenabrowser.net/datapages/).

### 2.2 scRNA-seq data processing and analysis

Two publicly available scRNA-seq datasets, GSE205506 and GSE132465, were included in the analysis. From GSE205506, 17 colorectal tumor specimens with deficient mismatch repair/microsatellite instability-high (dMMR/MSI-H) status were selected; these samples were collected following neoadjuvant PD-1 blockade therapy (toripalimab). The GSE132465 dataset included tumor samples from 23 colorectal cancer (CRC) patients who underwent surgical resection without prior treatment (4 MSI-H and 19 microsatellite-stable [MSS] cases). Data processing and analysis were performed using Seurat (v5.0.2) (15). Cells with fewer than 300 detected genes and genes expressed in fewer than 5 cells were removed. The data were normalized using the LogNormalize method (scaling factor = 10,000), and the 2,000 most variable genes were identified using the variance-stabilizing transformation (vst) method. The dataset was scaled, and principal component analysis (PCA) was carried out using these variable genes. Batch effects between the two datasets were corrected using the Harmony algorithm (v1.0) (16), with orig.ident specified as the batch variable. Harmony was run with default parameters, using the top 30 principal components as input. Convergence of integration was assessed visually, and the effectiveness of batch correction was evaluated through UMAP plots before and after integration (**Supplementary Figures S1A**, **2A**). Cell clustering was based on the first 30 Harmony-corrected principal components. A resolution of 0.1 was used to identify major cell types, and a resolution of 2.0 was applied for sub-clustering of T cells, B cells, and myeloid cells to define finer subpopulations. Dimensionality reduction for visualization was performed using Uniform Manifold Approximation and Projection (UMAP), with Barnes-Hut t-Distributed Stochastic Neighbor Embedding (t-SNE) used where appropriate. Cell types were annotated based on established canonical marker genes. 

### 2.3 Bulk transcriptome data processing and analysis

Gene expression levels in the TCGA-COAD dataset were normalized to TPM values and log-transformed using log2(TPM + 1). Microarray data from the GSE39582 dataset (14), obtained via the Affymetrix Human Genome U133 Plus 2.0 Array, underwent log2 transformation as well. Only patients with available survival data, excluding those with survival times shorter than 30 days, were included in the analysis.

### 2.4 Differential expression and pathway enrichment analysis

DEGs between NR vs R and high AA vs low AA groups in the scRNA-seq data were detected using Seurat’s FindMarkers function (default parameters: log2 fold change threshold = 0.1). Adjusted p-values were calculated for each gene. GO enrichment analysis of upregulated and downregulated significant DEGs (adjusted *P* < 0.05 and log2 fold change > 0 or < 0, respectively) was conducted separately using the clusterProfiler package (v4.13.0) (17) to identify significantly enriched biological processes. The enrichment score was determined by the proportion of DEGs within a given gene set. Gene set enrichment analysis (GSEA) was conducted via the GSEABase package (v1.66.0) (18), with genes ranked by fold change. Pathways with an adjusted *P* < 0.05 were deemed significantly enriched, with results visualized as bar plots.

### 2.5 Identifying of genes for AA score development

A total of 142 amino acid metabolism-related genes were retrieved from the MsigDB based on the GSEA results for non-response (NR) and response (R) groups in the epithelial cells from the GSE205506 dataset. These 142 genes were then intersected with the epithelial differentially expressed genes (DEGs) between the NR and R groups, resulting in the identification of 31 genes. These 31 genes were subsequently used to construct the amino acid (AA) score using the AUCell_calcAUC function from the AUCell package (v1.26.0) (19).

### 2.6 Patient classification into low and high AA groups

From the epithelial cell cluster of the GSE132465 dataset (13), a total of 17,455 cells were selected. The AA score for each epithelial cell was calculated using the 31 amino acid-related genes identified earlier, with the AUCell_calcAUC function from the AUCell package (v1.26.0) (19). The mean AA score for each patient was then computed to reflect the patient’s amino acid metabolism activity. Using the median AA score as a cutoff, 23 patients were divided into two groups: 12 in the low AA group and 11 in the high AA group.

### 2.7 Scoring of cell properties

Exhaustion and angiogenesis signatures, representing the functions of cell sub-clusters, were obtained from published studies (20, 21). Gene set variation analysis (GSVA) was performed to score each cell based on these gene sets using the GSVA package (v1.52.3) (22).

### 2.8 Evaluation of cell differentiation levels

Differentiation potential of immune cell subsets was assessed using CytoTRACE (v0.3.3) (23), which infers cellular developmental potential based on transcriptional diversity. Higher CytoTRACE scores indicate less differentiated states (e.g., stem-like or memory-like), while lower scores reflect more differentiated cells. The analysis was applied to T cell subclusters in GSE132465.

### 2.9 Cell-cell communication analysis

Intercellular communication networks for the high AA and low AA groups, based on 23 CRC samples from the GSE132465 scRNA-seq dataset, were inferred, visualized, and compared using the CellChat package (v2.1.2) (24) and prior knowledge from CellChatDB.

### 2.10 Estimation of immune cell infiltration from bulk RNA-seq data

Immune cell infiltration from bulk RNA-seq data was assessed using CIBERSORT (v0.1.0) (25). Immune cell abundance for each sample was estimated based on a reference set of 22 immune cell subtypes with 1,000 permutations.

### 2.11 Amino acid-related risk score construction

The risk score was developed from the TPM profile of CRC patients with complete survival data in the TCGA-COAD training cohort. The dataset was randomly split into training and test cohorts using the ‘caret’ package (v6.0-94). Univariate Cox analysis first identified survival-associated gene from DEGs in the scRNA-seq dataset (GSE132465) correlated with the AA score (r > 0.5, *P* < 0.05). LASSO regression then selected key genes, followed by multivariate Cox analysis to establish the prognostic model. The risk score was computed as: *risk score* = ∑^*n*^_i_ *Coef*(*gene_i_*) ∗ *Expression(gene_i_)*, where Coef(gene_i_) represents the multivariate Cox regression coefficient, Expression(gene_i_) is the corresponding gene expression, and n is the number of selected genes. Specifically, the amino acid-related risk score formula is: risk score = (-0.9095597 × MAPKAPK3) + (0.6674284 × RBM17) + (-0.4121224 × DHFR) + (-0.5263833 × MCCC2) + (1.3370698 × ARPC5L) + (-1.0453494 × ALG14).

### 2.12 Drug response prediction

Drug responses in TCGA-COAD patients were estimated using the oncoPredict package (v1.2) (26). The half-maximal inhibitory concentration (IC50) of each drug was determined based on bulk RNA-seq gene expression data and prior knowledge from the Genomics of Drug Sensitivity in Cancer (GDSC) database.

### 2.13 Cell culture

The murine colorectal cancer cell lines MC38 and CT26 were obtained from the ATCC and BMCR. MC38 and CT26 cells were cultured in DMEM (Hyclone) and RPMI-1640 (Gibco) medium supplemented with 10% fetal bovine serum (Corning), respectively. Both cell lines were confirmed mycoplasma-free using Mycolor One-Step Mycoplasma Detector (Vazyme) and maintained at 37°C in a 5% CO_2_ incubator.

### 2.14 siRNA transfection

Small interfering RNAs (siRNAs) from GenePharma were transfected into sub-confluent cells using the DharmaFECT-1 transfection reagent (Dharmacon). Transfections were performed in six-well plates, where 30% confluent CRC cells were treated with 5 µL of 20 µM siRNA and 5 µL of DharmaFECT-1 reagent in Opti-MEM medium (Gibco). A nonspecific siRNA served as a negative control. The sequences of siRNAs targeting Psat1 and Shmt2 are provided in **Supplementary Table S4**.

### 2.15 Reverse transcription-quantitative PCR analysis

Total RNA was extracted using the RNAsimple Total RNA kit (TIANGEN) and reverse-transcribed with Takara reagents and oligo(dT) primers. Quantitative PCR was performed on an ABI Prism 7900HT Sequence Detection System (Applied Biosystems, USA) using SYBR Premix Ex Taq II (Takara). Gene expression was quantifed via the 2^−ΔΔCT^ method, with ACTB as the normalization control. Primers sequences used in this study are listed in **Supplementary Table S4**.

### 2.16 Western blotting

Cells were lysed in RIPA buffer (Epizyme) supplemented with a protease and phosphatase inhibitor cocktail (MCE). Proteins were separated via SDS-PAGE and immunoblotted. Primary antibodies included anti-Psat1 (1:2000, Proteintech) and anti-Shmt2 (1:2000, Proteintech), and peroxidase-conjugated anti-rabbit (1:5000, Proteintech) was used as the secondary antibody. Bands were detected with an ECL substrate (Epizyme) and scanned on a ChemiDoc™ MP Imaging System (Bio-Rad).

### 2.17 Cell viability assays

2×10^3^ murine colorectal tumor cells were seeded in 96-well plates and cultured for 24 hours. Cell viability was then assessed by Cell Counting Kit 8 (DOJINO) following the manufacturer’s protocol.

### 2.18 CD8^+^ T cell and tumor cell co-culture assay

Naive CD8^+^ T cells were isolated from the spleens of C57BL/6 and BALB/c mice using the EasySep mouse CD8^+^ T cell isolation kit (STEMCELL) according to the manufacturer’s protocol and were then immediately activated with anti-CD3/CD28 antibody (Biolegend) in RPMI-1640 (Gibco) containing 10 ng/mL mouse IL-2 (MCE), 10% fetal bovine serum (Corning) and 1% Pen Strep Solution (Gibco). T cells were stimulated *in vitro* for 2 days before being co-cultured with tumor cells. 1 × 10^5^ MC38 or CT26 cells were seeded into 24-well plates with RPMI-1640 complete medium. Activated CD8^+^ T cells were co-cultured with tumor cells at a 1:1 ratio for 24 hours.

### 2.19 Intracellular cytokine staining and flow cytometry

After co-culturing for 24 hours, CD8^+^ T cells were collected and stimulated with Leukocyte Activation Cocktail (BD) for 4 hours at 37°C. After washing, cells were sequentially stained with Fixable Viability Stain 780 (BD) and anti-CD8 (BD) for 15 minutes at room temperature, then fixed and permeabilized using the Fixation/Permeablization Kit (BD) at 4°C for 30 minutes. After two washes with staining buffer, anti-Granzyme B (Biolegend) antibodies were added and incubated for 1 hour at 4°C. Samples were analyzed via flow cytometry, and results were processed via FlowJo software (v10.10.0).

### 2.20 Statistical analysis

All analyses were conducted in R (v4.4.0). Group differences were assessed using Wilcoxon rank-sum test or unpaired two-tailed t-test. Kaplan-Meier survival curves were evaluated with the log-rank test. Correlations were determined using Spearman’s test. Statistical significance was set at *P* < 0.05, with levels indicated as: * *P* < 0.05, ** *P* < 0.01, *** *P* < 0.001, **** *P* < 0.0001 and ns for not significant (*P* > 0.05).

## 3 Results

### 3.1 Epithelial cell cluster in PD-1 blockade-resistant CRC patients exhibits elevated amino acid metabolism

**Figure 1:**
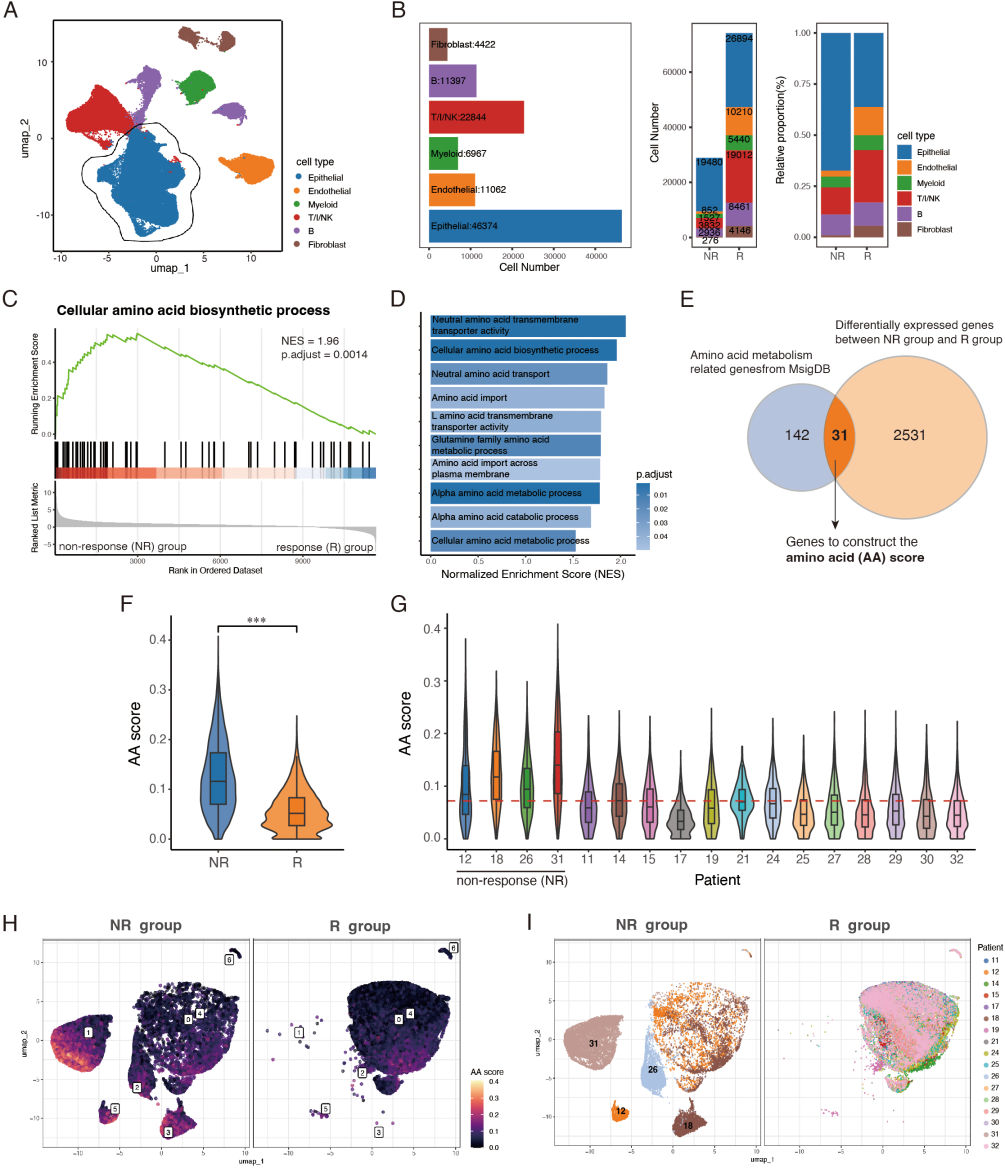
Epithelial cell cluster in PD-1 blockade-resistant CRC patients exhibits elevated amino acid metabolism. **(A)** UMAP visualization of 103,066 cells from 17 CRC patients, colored by cell types. **(B)** Bar plots showing identified cell types’ number (left) in sum, cell number (middle) and relative proportion (right) of 6 major cell types across NR and R groups. **(C, D)** GSEA analysis of metabolic pathways enriched in NR group. **(E)** Venn diagram of epithelial DEGs and amino acid metabolism related genes from MsigDB. **(F)** Violin-boxplots showing AA scores between NR and R groups. **(G)** Violin-boxplots of AA scores among 17 patients, red horizontal dashed line representing the median value of AA scores of all epithelial cells. **(H, I)** UMAP plots of epithelial cells, split by group, colored by AA score and patients, respectively. T/I/NK, T cells/innate lymphocytes/NK. NR, non-response. R, response. AA score, amino acid score. ***, *P* < 0.001.

To investigate the impact of tumor metabolic features on immune responses, we analyzed a single cell RNA sequencing (scRNA-seq) dataset GSE205506 from 17 colorectal cancer (CRC) patients who underwent neoadjuvant PD-1 blockade therapy (toripalimab) (12). Among these patients, 13 achieved a pathological complete response (R), while 4 were classified as non-responders (NR). After quality control and preprocessing, 103,066 cells were identified and classified into six major cell types: T/I/NK cells (T cells/innate lymphocytes/NK cells), B cells, myeloid cells, epithelial cells, endothelial cells, and fibroblasts, based on the expression of well-established marker genes (**Figure 1A**; **Supplementary Figure S1B**). Although tumor-infiltrating immune cells were more abundant in the R group compared to the NR group, epithelial cells remained the largest cluster in both groups, comprising approximately 50% of all cells analyzed (**Figure 1B**). We then isolated the epithelial cell cluster and conducted Gene Set Enrichment Analysis (GSEA) to explore metabolic pathway differences between the two groups. The enrichment analysis revealed that epithelial cells in the NR group exhibited increased amino acid metabolic activity, including upregulation of amino acid metabolic processes and amino acid transporter activity (**Figures 1C, D**).

To quantify amino acid metabolic activity, we developed an Amino Acid (AA) score using AUCell algorithm. The score was based on a gene set created by intersecting differentially expressed genes between the NR and R groups with amino acid-related genes from the Molecular Signatures Database (MSigDB) (**Figure 1E**). The resulting gene set comprises 31 genes, roughly half of which encode amino acid metabolic enzymes or transporters. Additionally, 9 genes are involved in energy metabolism and redox regulation, with amino acids serving as substrates for energy production and precursors to maintain redox homeostasis (27). 5 genes linked with purine and pyrimidine metabolism, presenting downstream of amino acid metabolism (28), and the oncogene MYC, which plays a pivotal role in coordinating metabolic changes to support rapid cell proliferation (29), is also included. (**Supplementary Table S1**).

Next, we performed sub-cluster analysis of epithelial cells and calculated the AA score for each cell. The NR group showed significantly higher AA scores compared to the R group (**Figure 1F**), and the mean AA scores of all 4 NR patients were above the median value (**Figure 1G**). While substantial heterogeneity was observed both between and within the NR and R groups, epithelial cells mainly from NR patients (sub-cluster 1, 2, 3 and 5) exhibited higher AA score (**Figures 1H, I**). We also assessed the AA scores across other major cell types and found that the increased amino acid metabolic activity was most pronounced in the epithelial cell cluster of the NR group (**Supplementary Figures S1C, D**). These findings suggest a strong association between elevated epithelial AA scores and the failure of PD-1 blockade therapy, highlighting upregulated amino acid metabolism as a potential metabolic hallmark of a suppressed tumor immune microenvironment.

### 3.2 Amino acid score differentiates immune microenvironments in CRC

**Figure 2:**
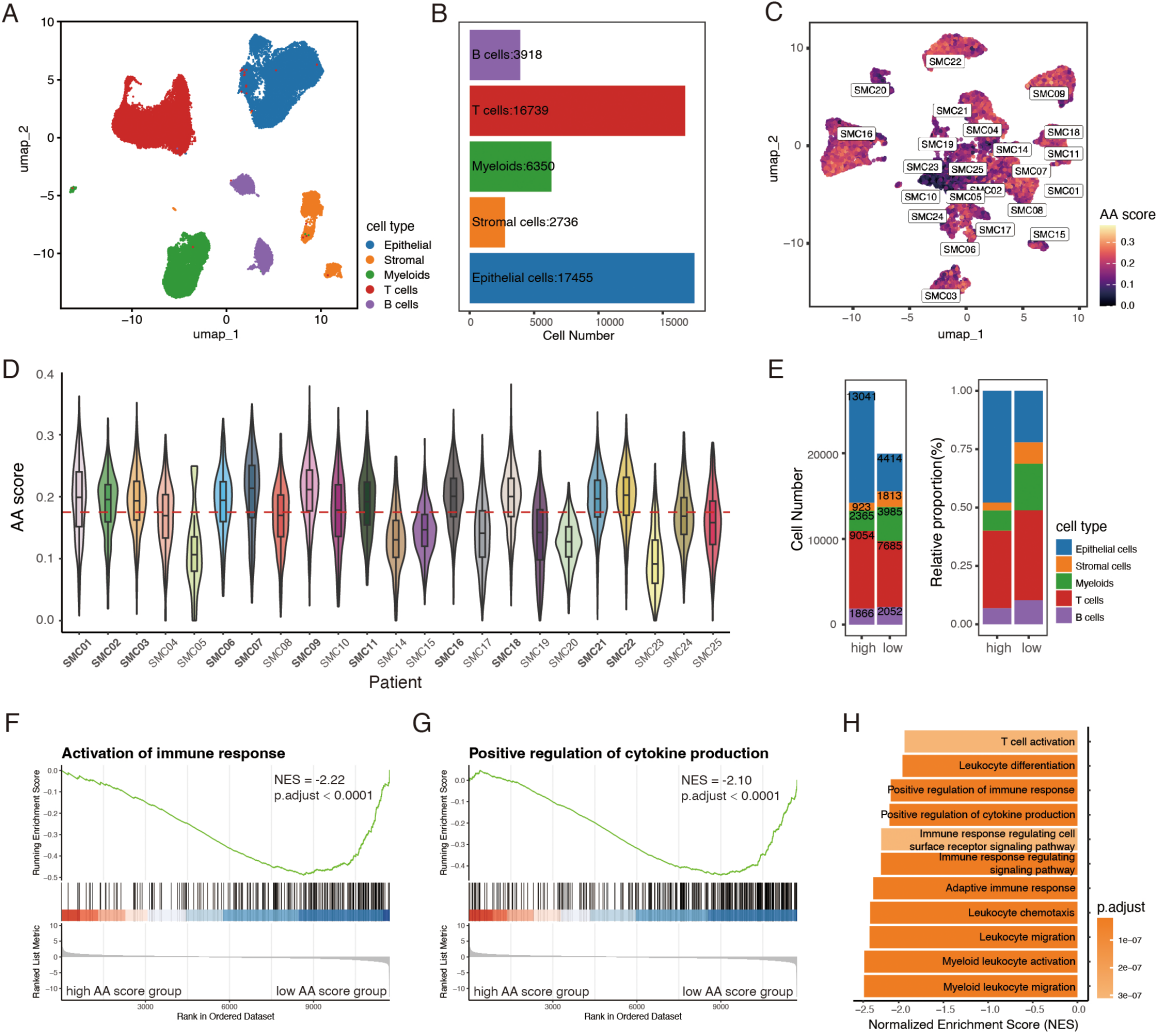
Amino acid (AA) score differentiates immune microenvironments in CRC. **(A)** UMAP plot of 47,198 cells from 23 CRC samples in GSE132465 dataset. **(B)** Bar plot showing the cell number of 5 major cell types. **(C)** UMAP visualization of 17,455 epithelial cells, colored by AA score, labeled by patient. **(D)** Violin-boxplot showing epithelial AA score for each patient, red horizontal dashed line representing the median AA score across 23 tumor samples. **(E)** Bar plots of number and relative proportion of each cell type between the high and low AA groups. **(F-H)** GSEA analysis showing pathways enriched in the low AA group. AA, amino acid.

An effective anti-tumor immune response is a multi-step process requiring the coordination of diverse immune cells (3). Thus, characterizing immune cell abundance, function, and properties within the tumor microenvironment is crucial for identifying determinants of PD-1 therapy response. To investigate how amino acid metabolism alterations shape the tumor microenvironment, a total of 47,198 cells from 23 CRC patients in the GSE132465 dataset were included for further analysis. Epithelial cells, stromal cells, T cells, B cells and myeloid cells were identified as 5 broad cell types (**Figures 2A, B**; **Supplementary Figure S2B**). Mast cells, represented by only three cells, were excluded due to their minimal presence in the samples. We calculated the amino acid (AA) score for each epithelial cell using 31 previously identified genes (**Figure 2C**) and analyzed the average AA activity within each patient. Based on the median value of patients’ AA scores, the 23 CRC samples were divided into two groups: 11 samples were classified as the high AA group, and 12 as the low AA group (**Figure 2D**). While the overall cell composition showed minimal variation between the groups, the high AA group exhibited a higher number and percentage of epithelial cells compared to the low AA group (**Figure 2E**).

To get an overview of the differences in biological activity between the two groups, we performed GSEA analysis. The results aligned with our previous findings, revealing a stronger immune response in the low AA group. Pathways associated with immune activation, cytokine production, and leukocyte migration, differentiation, and activation were significantly enriched in the low AA group (**Figures 2F-H**). In summary, these findings suggest that amino acid metabolism of epithelial cells may serve as a potential classifier for distinct immune microenvironments in CRC.

### 3.3 Variations in cell-cell interactions and signaling pathways across amino acid score levels

**Figure 3:**
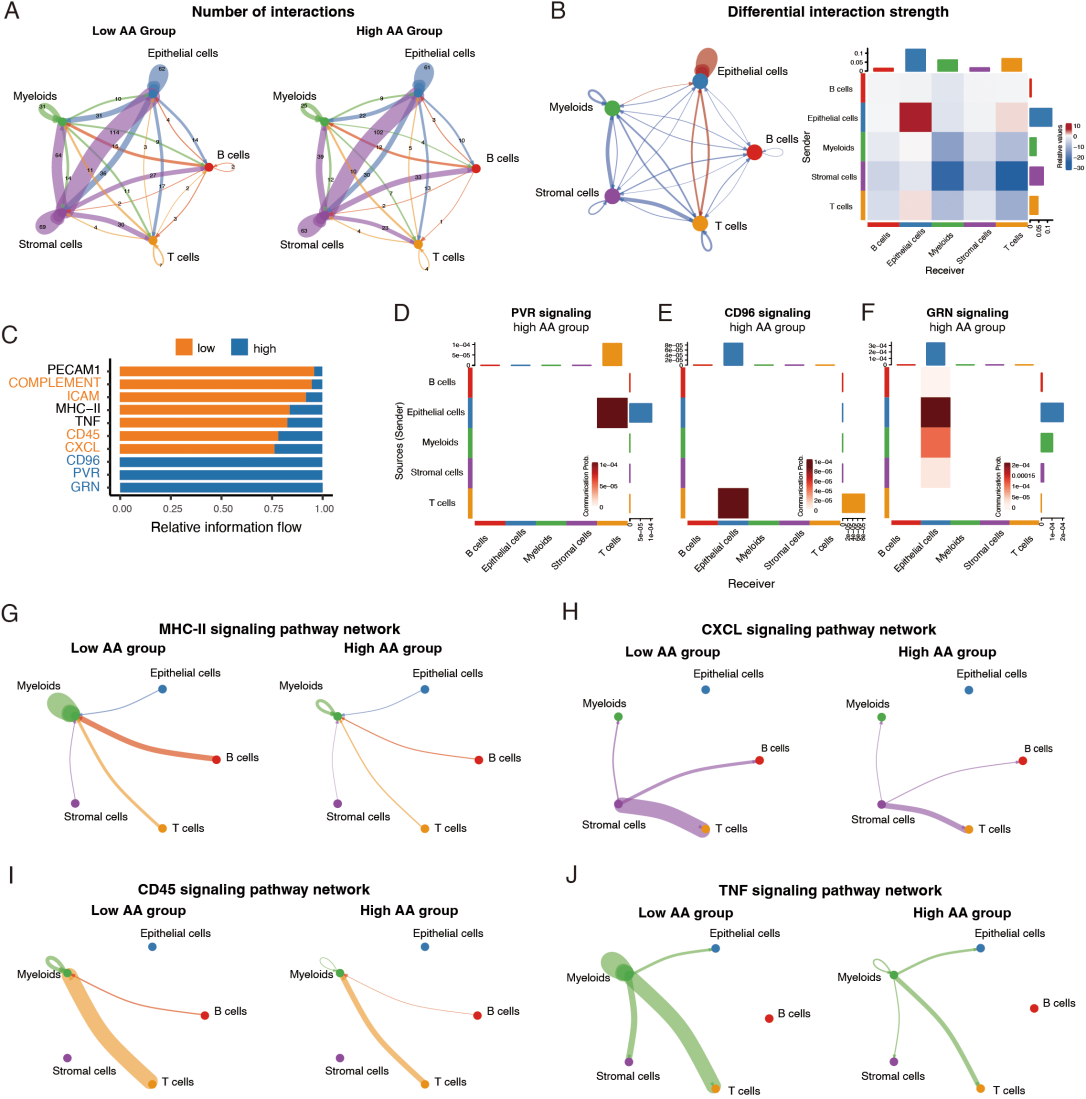
Variations in cell-cell interactions and signaling pathways across amino acid (AA) score levels. **(A)** Circle plots showing numbers of cell-cell interactions inferred by CellChat in two groups. **(B)** Circle plot and heatmap showing differential interaction strength across two groups. Red and blue indicating interactions upregulated and downregulated in high AA group, respectively. **(C)** Bar plots of relative signaling flow among two groups. **(D-F)** Heatmap plots of signaling pathways only present in high AA group. **(G-J)** Circle plots showing differences of specific signaling pathways networks between high AA group and low AA group. AA, amino acid.

Growing evidence suggests that the dynamic interactions among diverse cells in the tumor microenvironment collectively orchestrate malignant progression (7). Thus, we used CellChat to infer cell-cell communication networks and compare interaction patterns between the two groups. The low AA group demonstrated a higher frequency of nearly all types of intercellular interactions compared to the high AA group (**Figure 3A**). While most interactions were stronger in the low AA group, the high AA group displayed notably enhanced signaling within the epithelial cell cluster and a modest increase in interactions between epithelial cells and T cells (**Figure 3B**).

To further explore the factors influencing cell-cell communication networks, we examined the differences in specific signaling pathways among the two groups. In the high AA group, three immune-suppressive pathways, CD96, PVR, and GRN signaling, were uniquely present (**Figures 3C–F**). Specifically, epithelial cells in this group acted as the source of Poliovirus receptor (PVR or CD155) signaling (30), which dampens the anti-tumor activity of T cells (**Figure 3D**) (31). Similarly, CD96 signaling, an immune checkpoint receptor pathway (32), was detected between epithelial cells and T cells (**Figure 3E**). Granulins (GRN) signaling, known to promote the immunosuppressive M2 macrophage phenotype (33), was observed between epithelial cells and myeloid cells (**Figure 3F**). In contrast, the low AA group exhibited enhanced activity in pathways associated with antigen presentation (MHC-II signaling) (34), immune cell adhesion and recruitment (ICAM, PECAM1, and CXCL signaling) (35), and immune activation (complement, CD45 and TNF signaling) (36, 37) (**Figures 3C, G–J**; **Supplementary Figures S3A-C**). Specifically, MHC-II signaling from B cells and T cells to myeloid cells, along with interactions within myeloid cells, was more pronounced in the low AA group (**Figure 3G**). Elevated CXCL signaling from stromal cells may have facilitated the recruitment of additional T cells, B cells, and myeloid cells to the tumor microenvironment (**Figure 3H**). CD45 signaling, observed between myeloid cells, T cells, and B cells, likely contributed to effective immune responses (**Figure 3I**). Additionally, myeloid cells acted as senders of Tumor Necrosis Factor (TNF) signaling, with T cells, epithelial cells, stromal cells, and myeloid cells themselves as recipients, demonstrating a more prominent interaction in the low AA group (**Figure 3J**).

Collectively, these findings reveal a fundamental shift in the immune landscape across the two groups, with the high AA group dominated by immune-suppressive mechanisms and the low AA group displaying a stronger immune activation profile.

### 3.4 Enhanced T cell-mediated immune response in the low amino acid group

**Figure 4:**
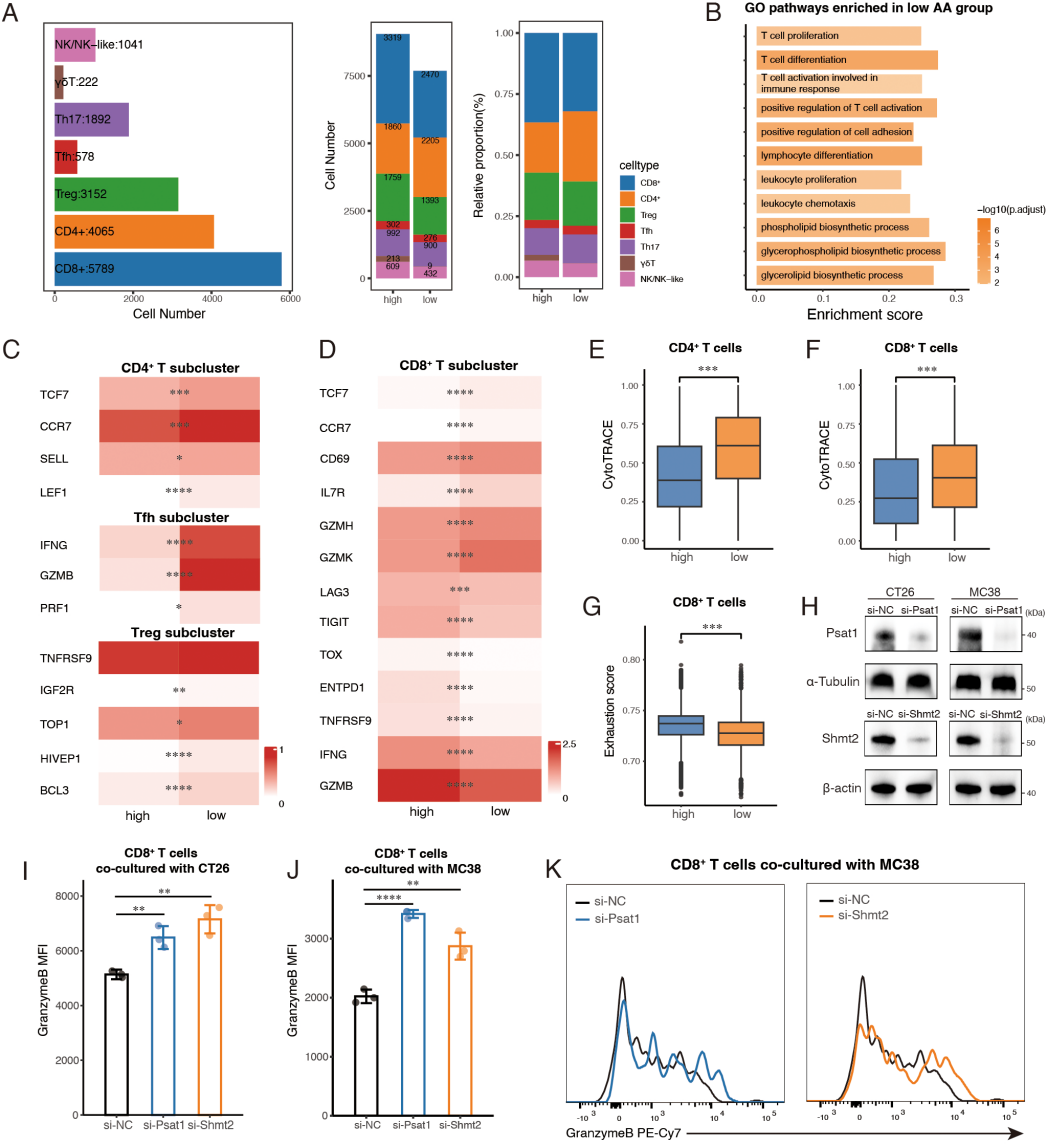
Enhanced T cell-mediated immune response in the low amino acid (AA) group. **(A)** Bar plots showing cell number of T sub-clusters (left) in total, cell number (middle) and relative proportion (right) of subpopulations of T cells between high AA and low AA groups. **(B)** GO analysis of pathways enriched in low AA group. **(C, D)** Heatmaps showing the expression of representative marker genes across T cell subsets between high AA and low AA groups. Color intensity represents expression levels. **(E, F)** Boxplots showing CytoTRACE scores of CD4^+^ and CD8^+^ T cells among two groups. **(G)** Boxplots of exhaustion scores calculated by GSVA of CD8^+^ T cells between high AA group and low AA group. **(H)** Protein levels were confirmed by Western blotting after siRNA transfection. **(I, J)** Quantification of Granzyme B mean fluorescence intensity (MFI) in CD8^+^ T cells co-cultured with CT26 and MC38 colorectal cancer cells following the indicated treatment (n=3). **(K)** Representative histogram showing Granzyme B fluorescence intensity in CD8^+^ T cells co-cultured with MC38 cells. AA, amino acid. Treg, regulatory T cell. Tfh, follicular helper T cell. Th17, T helper cell 17. *, *P* < 0.05; **, *P* < 0.01; ***, *P* < 0.001; ****, *P* < 0.0001.

To examine immune cell subtype composition and functional differences between the two groups, we separately re-clustered and analyzed three major immune cell types, T cells, B cells and myeloid cells. A total of 16,739 T cells were categorized into seven subclusters based on canonical markers (**Supplementary Figures S4A, B**). Within the CD4^+^ T cells, distinct clusters were identified for Treg (regulatory T cell), Tfh (follicular helper T cell), and Th17 (T helper cell 17), each defined by specific transcriptional markers (**Supplementary Figure S4B**). The remaining CD4^+^ T cells were grouped into a single CD4^+^ subcluster. Additional clusters included CD8^+^ T cells, γδT cells, and NK/NK-like cells (**Supplementary Figure S4B**). While the overall composition of T cell subtypes was consistent between the groups, γδT cells were predominantly observed in the high group (**Figure 4A**).

We first conducted differential expressed gene (DEG) analysis on the major T cell clusters between the two groups, followed by Gene Ontology (GO) enrichment analysis. Pathways associated with a more active immune response, including ‘T cell activation involved in immune response’, ‘T cell differentiation’ and ‘T cell proliferation’ were enriched in the low AA group (**Figure 4B**). Notably, lipid metabolism pathways, specifically glycerophospholipid, glycerolipid, and phospholipid biosynthesis, were also highly ranked in the low AA group (**Figure 4B**). Given the essential role of these pathways in supporting T cell function during anti-tumor immunity (38, 39), we propose that their enrichment may contribute to the observed cytotoxic phenotype in T cells from the low AA group by facilitating TCR signaling, enhancing energy storage, and promoting anti-tumor immune responses.

Given the central role of CD4^+^ and CD8^+^ T cells in anti-tumor immunity and their predominance in our dataset, we focused our analysis on these subsets. UMAP visualization confirmed that naïve T cells were primarily composed of CD4^+^ T cells (**Supplementary Figure S4C**). In the low AA group, CD4^+^ T cells showed increased expression of naïve and memory-associated markers, including CCR7, TCF7, and LEF1 (40) (**Figure 4C**), and exhibited lower differentiation status as indicated by higher CytoTRACE scores (**Figure 4E**). Further analysis of marker gene expression revealed that Tfh and Treg cells in the low AA group expressed higher levels of markers characteristic of IFNG^+^ Tfh cells and TNFRSF9^+^ Treg cells (**Figure 4C**), which are identified as key tumor-reactive subclusters (20). These findings suggest that CD4^+^ T cell subsets in the low AA group may have enhanced anti-tumor activity.

In the CD8^+^ compartment, the low AA group displayed higher expression of naïve, early activation, and cytotoxic markers, including TCF7, CCR7, CD69, GZMK, and GZMH (**Figure 4D**). Additionally, elevated expression of GZMK and IL7R, associated with effector memory (Tem) and memory T cells (Tm), respectively, suggests an enrichment of activated and memory-like populations. A subset of GZMK^+^ early Tem cells also co-expressed CXCR5 or TCF7, indicating a transitional phenotype between naïve and terminally differentiated states (20, 41). Consistent with these patterns, CytoTRACE analysis showed that CD8^+^ T cells in the low AA group had greater differentiation potential (**Figure 4F**). By contrast, the high AA group exhibited elevated expression of exhaustion markers (20), including TOX, TIGIT, LAG3, and TNFRSF9, along with higher levels of ENTPD1, IFNG, and GZMB, indicating a predominance of terminally exhausted CD8^+^ T cells (**Figure 4D**). GSVA analysis further confirmed that CD8^+^ T cells in this group had significantly higher exhaustion scores (20) (**Figure 4G**). Collectively, these results suggest that CD8^+^ T cells in the low AA group are enriched for transitional states between naïve/memory and terminal exhaustion, whereas the high AA group contains a larger population of terminally exhausted CD8^+^ T cells.

To further investigate the role of tumor metabolism-driven immune evasion, we chose two key enzymes in serine/glycine metabolism (42), PSAT1 and SHMT2, which ranked among the top five differentially expressed genes in the AA score gene set across both scRNA-seq datasets (**Supplementary Table S3**) for *in vitro* co-culture experiments with mouse spleen-derived CD8^+^ T cells. Small interfering RNAs (siRNAs) were used to individually knock down Psat1 and Shmt2 in two murine colorectal cancer cell lines (CT26 and MC38), with knockdown efficiency confirmed by RT-qPCR (**Supplementary Figure S4D**) and Western blot (**Figure 4H**). To validate metabolic perturbations following knockdown, we performed qPCR of key downstream metabolic genes (Shmt2, Mthfd2, Tyms, Dhfr, Gart, Mat2a, Gclc, and Gpx4) in CT26 and MC38 cells. Knockdown of Psat1 or Shmt2 led to reduced expression of multiple metabolic genes, confirming pathway disruption (**Supplementary Figure S4E**). A CCK8 assay showed that depleting Psat1 or Shmt2 did not significantly impact cell viability within 24 hours (**Supplementary Figures S4F**). However, co-culturing CD8^+^ T cells with Psat1- or Shmt2-deficient colorectal cancer cells showed enhanced cytotoxic activity, as indicated by increased Granzyme B levels detected via flow cytometry (**Figures 4I-K**; **Supplementary Figure S4G**). Together, these findings suggested that T cells in microenvironments with lower epithelial amino acid metabolism activity exhibit enhanced anti-tumor functions.

### 3.5 Distinct traits of tumor-infiltrating B and myeloid cells between amino acid groups

**Figure 5:**
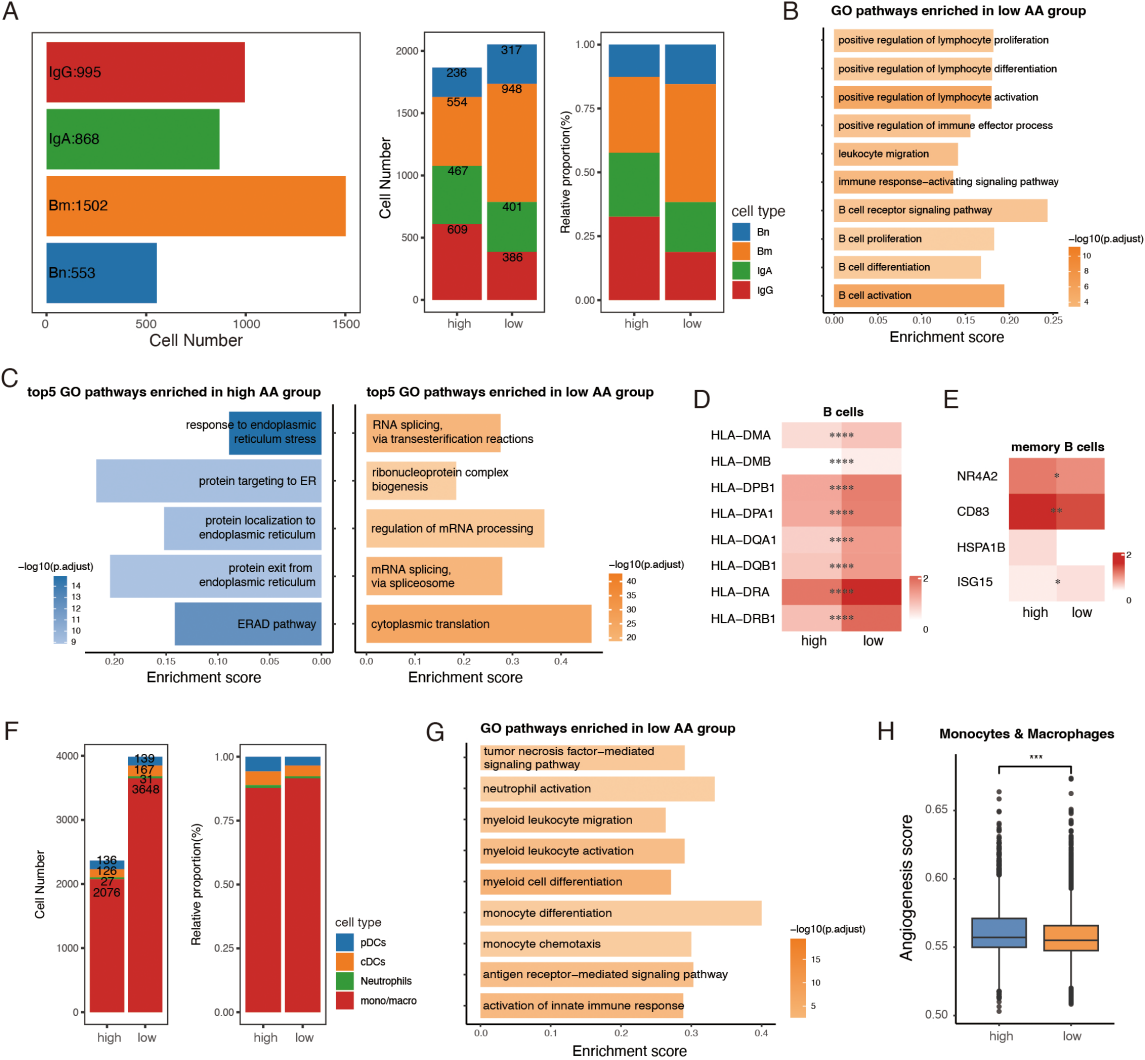
Distinct traits of tumor-infiltrating B and myeloid cells between amino acid (AA) groups. **(A)** Bar plots showing numbers of B subclusters (left) in total, cell number (middle) and relative proportion (right) of subclusters of B cells between high AA and low AA groups. **(B)** GO analysis of immune-related pathways in low the AA group. **(C)** GO analysis of top 5 pathways enriched in high AA and low AA groups. **(D, E)** Heatmaps showing the expression of representative marker genes across B cell subsets between high AA and low AA groups. Color intensity represents expression levels. **(F)** Bar plots showing the number and relative proportion of subclusters of myeloid cells between the two groups. **(G)** GO analysis showing pathways enriched in low AA group. **(H)** Boxplots of angiogenesis scores of monocytes and macrophages among the two groups. AA, amino acid. Bn, naïve B cells. Bm, memory B cells. IgA, IgA^+^ plasma cells. IgG, IgG^+^ plasma cells. pDCs, plasmacytoid dendritic cells. cDCs, conventional dendritic cells. mono/macro, monocytes/macrophages. *, *P* < 0.05; **, *P* < 0.01; ***, *P* < 0.001; ****, *P* < 0.0001.

For the major B cell cluster, 3,918 cells were included in the downstream analysis. Classical CD19^+^ CD20^+^ B cells were divided into naïve and memory subpopulations based on their distinct expression patterns of IGHD, TCL1A, and TNFRSF13B (43) (**Supplementary Figures S5A, B**). Plasma cells were further classified into IgA^+^ and IgG^+^ subsets according to the expression levels of IGHA1, IGHA2, IGHG1, and IGHG3 (**Supplementary Figures S5A, B**). Notably, memory B cells were more abundant in the low AA group, whereas the high AA group exhibited a greater proportion of IgG^+^ plasma cells (**Figure 5A**).

To uncover functional differences, DEG and GO enrichment analyses were performed on all B cells and plasma cells between the two groups. Pathways associated with B cell activation, proliferation, differentiation, and B cell receptor (BCR) signaling were enriched in the low AA group, suggesting a more robust immune response (**Figure 5B**). Interestingly, pathway analysis revealed contrasting cellular activities: the major B cell cluster from the high AA group exhibited signs of endoplasmic reticulum (ER) stress (44) and protein degradation (**Figure 5C**). In contrast, B cells from the low-AA group showed increased protein translation activity (**Figure 5C**). Further analysis of B cell subclusters showed that multiple antigen-processing genes were significantly upregulated in the low AA group, reflecting enhanced antigen presentation capacity (**Figure 5D**). Notably, memory B cells with a stress-response signature have been associated with poor prognosis across various cancer types (43). In our data, stress-associated genes—NR4A2, CD83, and HSPA1B—were elevated in memory B cells from the high AA group, whereas ISG15, a marker of IFN-γ response, was more highly expressed in the low AA group (**Figure 5E**). These findings suggest that B cells in the low AA group may be more functionally equipped for anti-tumor responses, while those in the high AA group display features of cellular stress and dysfunction.

A pool of 6,350 myeloid cells was identified and categorized into four common major lineages: plasmacytoid dendritic cells (pDCs), conventional dendritic cells (cDCs), neutrophils and monocytes/macrophages (mono/macro), using canonical cell markers (45) (**Supplementary Figures S5C, D**). While the number of tumor-infiltrating myeloid cells was significantly lower in the high AA group compared to the low AA group, monocytes and macrophages accounted for over 80% of the myeloid population in both groups (**Figure 5F**). The dataset revealed a substantial presence of pro-inflammatory monocyte-like macrophages with high FCN1, S100A8, S100A9, and IL1B expression, alongside SPP1^+^ and C1QC^+^ macrophages, which are associated with pro-tumorigenic functions (**Supplementary Figure S5E**). Functional analysis highlighted that pathways promoting myeloid cell activation and differentiation were enriched in the low AA group (**Figure 5G**). In contrast, monocytes and macrophages in the high AA group displayed enhanced angiogenic activity, a feature linked to resistance against myeloid-targeted therapies, such as anti-CSF1R blockade (21) (**Figure 5H**).

Taken together, these findings demonstrated distinct characteristics of tumor-infiltrating B cells and myeloid cells between the two groups. Immune cells in the low AA group exhibited higher activation and differentiation levels, suggesting a more robust anti-tumor immune response.

### 3.6 Generation and validation of an amino-acid related risk score to reveal prognosis, immune characteristics and therapeutic implications

**Figure 6:**
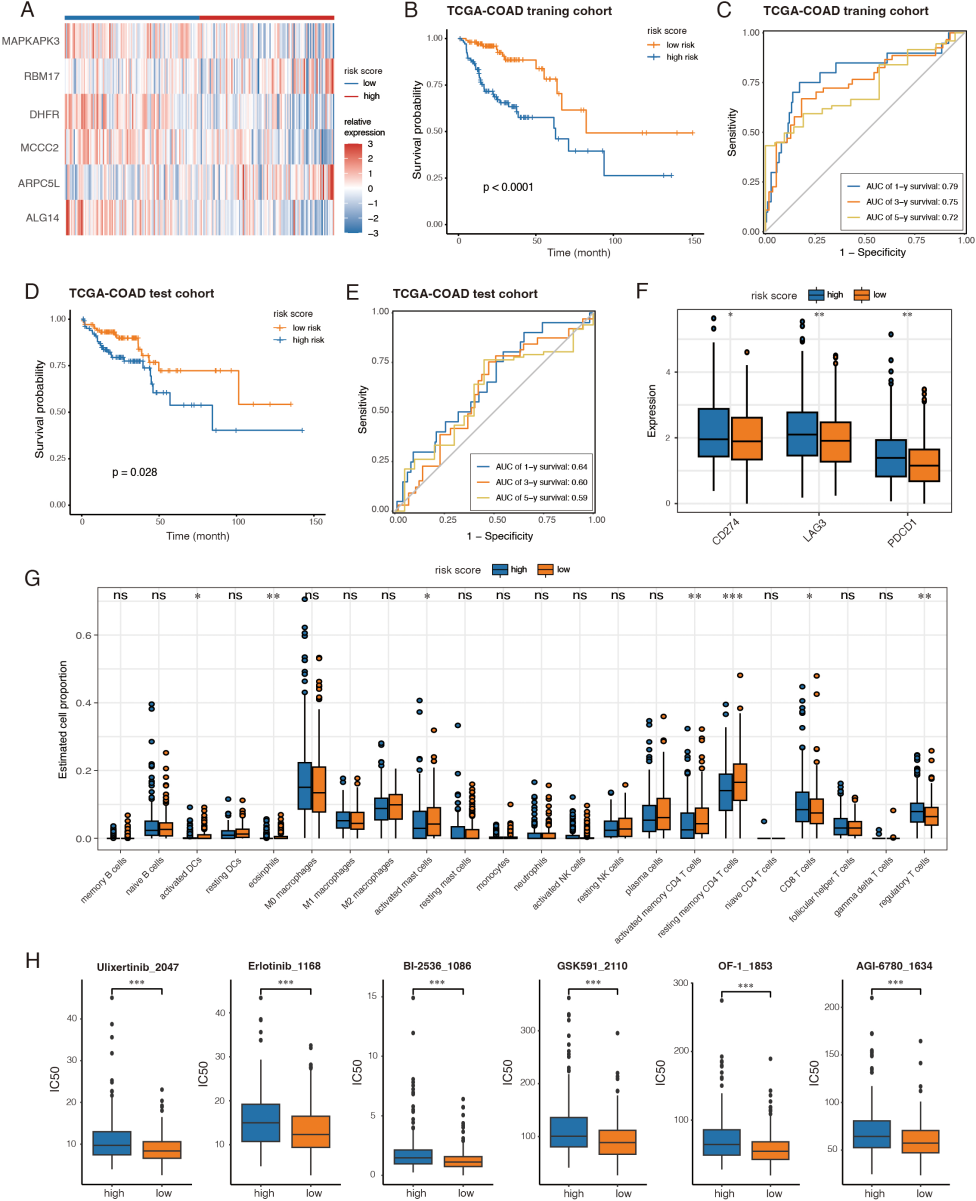
Generation and validation of an amino-acid related risk score to reveal prognosis, immune characteristics and therapeutic implications. **(A)** Heatmap displayed expression of the 6 genes between the two groups. **(B, D)** Kaplan-Meier analyses between high-risk groups and low-risk groups in the training and test cohorts, respectively. **(C, E)** Time-dependent ROC curves of risk score predicting the 1-, 3-, and 5-year overall survival (OS) of the patients from the training and test cohorts, respectively. **(F)** The expression of immune checkpoints between high-risk and low-risk groups from the TCGA-COAD cohort. **(G)** Boxplots showing tumor-infiltrating immune cell proportions estimated by CIBERSORT across two groups. **(H)** The results of drug sensitivity analysis between high-risk and low-risk groups. *, *P* < 0.05; **, *P* < 0.01; ***, *P* < 0.001; ns, not significant, *P* > 0.05.

To validate and expand previous findings using bulk RNA-seq data, we collected all differential expressed genes from GSE132465 between high AA and low AA groups, and selected those with expression correlated to the AA score. The TCGA-COAD dataset was evenly split into training and test cohorts, each containing half of the samples. Genes pooled from scRNA-seq data underwent univariate Cox analysis, identifying 69 genes significantly associated with survival (*P* < 0.05) in the TCGA training cohort. LASSO and multivariate Cox analyses further refined the selection to six key genes (MAPKAPK3, RBM17, DHFR, MCCC2, ARPC5L, and ALG14) (**Figure 6A**; **Supplementary Figures S6A-C**).

A risk score was computed for each patient using the expression levels and coefficients of the six genes. The training cohort was stratified into high- and low-risk groups based on the median score. Kaplan-Meier survival analysis revealed that high-risk patients had significantly shorter long-term survival (**Figure 6B**). The risk score demonstrated strong predictive accuracy for 1-, 3-, and 5-year survival, with probabilities exceeding 0.7 (**Figure 6C**). These findings were validated in both the TCGA-COAD test cohort and the GSE39582 dataset (14) (**Figures 6D, E**; **Supplementary Figures S6D, E**).

We also analyzed immune checkpoint gene expression across all TCGA-COAD patients. High-risk patients exhibited significantly elevated expression of PD1 (PDCD1), PDL1 (CD274) and LAG3 (**Figure 6F**). Additionally, immune cell proportions estimated by CIBERSORT revealed differences in tumor immune infiltration: while CD8^+^ T cells were more abundant in the high-risk group, low-risk patients exhibited higher levels of activated dendritic cells, CD4^+^ memory T cells, and fewer regulatory T cells (**Figure 6G**).

Drug sensitivity analysis was conducted using the oncoPredict package, integrating gene expression data with prior knowledge from the GDSC dataset. Among the drugs with the greatest IC50 differences, low-risk patients showed higher sensitivity to ERK inhibitor (Ulixertinib), EGFR inhibitor (Erlotinib), chromatin-modifying agents (OF-1, GSK591), and cell cycle regulators (BI-2536) (**Figure 6H**). Notably, high-risk patients were more resistant to AGI-6780 (**Figure 6H**), an IDH mutation-targeting inhibitor that disrupts the Warburg effect.

Together, the six-gene-based risk score effectively predicts prognosis, correlates strongly with immune checkpoint expression and immune cell infiltration, and may serve as a valuable tool to guide therapeutic strategies.

## 4 Discussion

In this study, we demonstrated that elevated amino acid metabolism in CRC epithelial cells contributes to an immunosuppressive tumor microenvironment and is associated with resistance to PD-1 blockade therapy. By leveraging scRNA-seq data, we quantified metabolic activity at the single-cell level and found that tumors with lower epithelial amino acid metabolism exhibited more robust immune responses, including enhanced activation and differentiation of tumor-infiltrating T, B, and myeloid cells. Additionally, we developed an amino acid metabolism-related risk score based on six genes, which correlated with immune infiltration, immune checkpoint expression, and patient prognosis. These findings highlight the critical role of epithelial amino acid metabolism in shaping tumor immunity and suggest potential metabolic vulnerabilities that could be exploited for therapeutic interventions.

Growing evidence supports the notion that metabolic reprogramming of amino acid pathways in cancer cells plays a crucial role in tumor progression by fostering an immune-suppressive microenvironment and facilitating immune evasion (46). For instance, glutaminase or methionine adenosyltransferase 2A deletion in mouse models enhanced T cell–mediated tumor control (47, 48). Cancer cells often overexpress SLC43A2, limiting methionine availability for T cells (10), and IDO1 hyperactivation promotes immunosuppression via kynurenine accumulation (49). These prior studies align with our findings, highlighting the key role of epithelial amino acid metabolism in modulating immune responses within the tumor microenvironment.

In efforts to translate these findings into clinical applications, several transcriptional signatures have been developed to predict immune infiltration and disease outcomes across various cancers (50–52). Many of these studies focus on specific amino acids, like branched-chain amino acids, or examine broader amino acid metabolism patterns in a pan-cancer context. While such approaches have identified metabolic gene sets linked to patient survival and treatment response, our study takes a more granular perspective by specifically interrogating epithelial cell metabolism at the single-cell level. Our findings indicate that epithelial amino acid metabolism is not only a marker of resistance to PD-1 blockade therapy but also an active driver of immune suppression—a facet that has not been fully explored in previous studies.

Despite these insights, several limitations should be noted. First, the small sample size of non-responders to neoadjuvant PD-1 blockade therapy limits statistical power and calls for cautious interpretation. However, we still observed that lower AA scores were associated with stronger immune activation within this subgroup, lending support to the robustness of our findings. Second, translating single-cell insights into bulk RNA-seq models presents inherent challenges. Bulk RNA-seq captures averaged signals from mixed cell populations, making it difficult to separate true gene expression changes from shifts in cell composition. In addition, technical differences between bulk and single-cell RNA-seq can complicate direct comparisons and influence model performance. These factors may explain discrepancies such as the differing patterns of CD8^+^ T cell infiltration observed between bulk and single-cell analyses. While our risk score model showed strong prognostic value and potential to guide immune profiling and therapy decisions, further validation in larger, independent cohorts and across diverse cancer types is essential. Third, our study relied on transcriptomic data, which, although informative, do not directly measure metabolic activity or metabolite levels. Incorporating metabolomics and functional assays would provide a more complete picture of how metabolic changes impact immune responses. Finally, the precise mechanisms by which amino acid metabolism shapes the immune landscape remain unclear, particularly regarding cell-cell interactions and metabolic competition. Future research should apply integrated multi-omics and *in vivo* functional studies to deepen understanding of these pathways.

In conclusion, our study reveals the impact of epithelial amino acid metabolism on the immune landscape in CRC, linking high metabolism to an immunosuppressive tumor microenvironment and potential resistance to immune checkpoint blockade. We also developed and validated a six-gene risk score, integrating scRNA-seq and bulk transcriptomic data for clinical risk stratification and treatment guidance. These findings highlight the influence of tumor metabolic reprogramming on immune escape, offer new perspectives for advancing immunotherapy, and lay the groundwork for future studies to clarify the underlying mechanisms.

## Data Availability

The single cell RNA-seq data and bulk mRNA arrays are accessible through the GEO database (https://www.ncbi.nlm.nih.gov/gds) under accession numbers GSE205506, GSE132465 and GSE39582. TCGA-COAD data was downloaded through UCSC Xena browser (https://xenabrowser.net/datapages/).

## Supplementary material

Supplementary Figure 1 Epithelial cell cluster in PD-1 blockade-resistant CRC patients exhibits elevated amino acid metabolism. Related to **Figure 1**. **(A)** UMAP plots of all cells grouped by samples before and after Harmony batch effect correction. **(B)** Dot plot showing average expression of known markers in indicated cell types. **(C)** UMAP plots of all cells colored by AA score calculated between NR and R groups. **(D)** Violin plots presenting each cell type’s AA score across NR and R groups. T/I/NK, T cells/innate lymphocytes/NK. AA score, amino acid score. NR, non-response. R, response.

Supplementary Figure 2 Amino acid (AA) score differentiates immune microenvironments in CRC. Related to **Figure 2**. **(A)** UMAP plots of all cells grouped by samples before and after Harmony batch effect correction. **(B)** Dot plot showing the average expression and percent expression of marker genes across cell types.

Supplementary Figure 3 Variations in cell-cell interactions and signaling pathways across amino acid (AA) score levels. Related to **Figure 3**. **(A-C)** Circle plots of differences of specific signaling pathways networks between high AA group and low AA group.

Supplementary Figure 4 Enhanced T cell-mediated immune response in the low amino acid (AA) group. Related to **Figure 4**. **(A)** UMAP visualization of cells from the major T cluster, colored by T cell subsets. **(B)** Dot plot of representative cell markers among each T subcluster; dot size represents abundance, and color represents expression level. **(C)** UMAP feature plot of naive marker gene expression of the major T cluster cells. **(D)** Gene expression levels validated by RT-qPCR after siRNA transfection. **(E)** qPCR validation of downstream metabolic genes in Psat1- and Shmt2-knockdown CRC cell lines. Significance was assessed by comparing si-Psat1 vs NC and si-Shmt2 vs NC. **(F)** Histograms of relative cell viability in CT26 and MC38 cells 24 hours post-seeding. **(G)** Representative histogram of Granzyme B fluorescence intensity in CD8^+^ T cells co-cultured with CT26 cells. *, *P* < 0.05; **, *P* < 0.01; ***, *P* < 0.001; ns, not significant, *P* > 0.05.

Supplementary Figure 5 Distinct traits of tumor-infiltrating B and myeloid cells between amino acid (AA) groups. Related to **Figure 5**. **(A)** t-SNE visualization of subtypes from major B cell cluster. **(B)** Dot plot showing the average expression and percent expression of marker genes across B cell subtypes. **(C)** t-SNE plot of subpopulations from myeloid cluster. **(D)** Dot plot showing the average expression and percent expression of marker genes across myeloid subtypes. **(E)** t-SNE feature plots of marker genes of myeloid cells. Bn, naïve B cells. Bm, memory B cells. IgA, IgA^+^ plasma cells. IgG, IgG^+^ plasma cells. pDCs, plasmacytoid dendritic cells. cDCs, conventional dendritic cells. mono/macro, monocytes/macrophages.

Supplementary Figure 6 Generation and validation of an amino-acid related risk score to reveal prognosis, immune characteristics and therapeutic implications. Related to **Figure 6**. **(A)** LASSO Cox regression analysis of the association between deviance and log(λ). **(B)** LASSO Cox regression analysis of the association between coefficients of genes and log(λ). **(C)** Forest plot showing hazard ratio of 6 selected genes. **(D, E)** The OS discrepancy between the high-risk and low-risk groups and the ROC curve of risk score predicting the 1-, 3-, and 5-year OS using the GSE39582 dataset.
