# Supplementary figures and images for: Single-cell and bulk transcriptome analyses reveal elevated amino acid metabolism promoting tumor-directed immune evasion in colorectal cancer
Source: Front Immunol. 2025 May 22;16:1575829. doi: 10.3389/fimmu.2025.1575829 (PMC12137356; doi:10.3389/fimmu.2025.1575829)

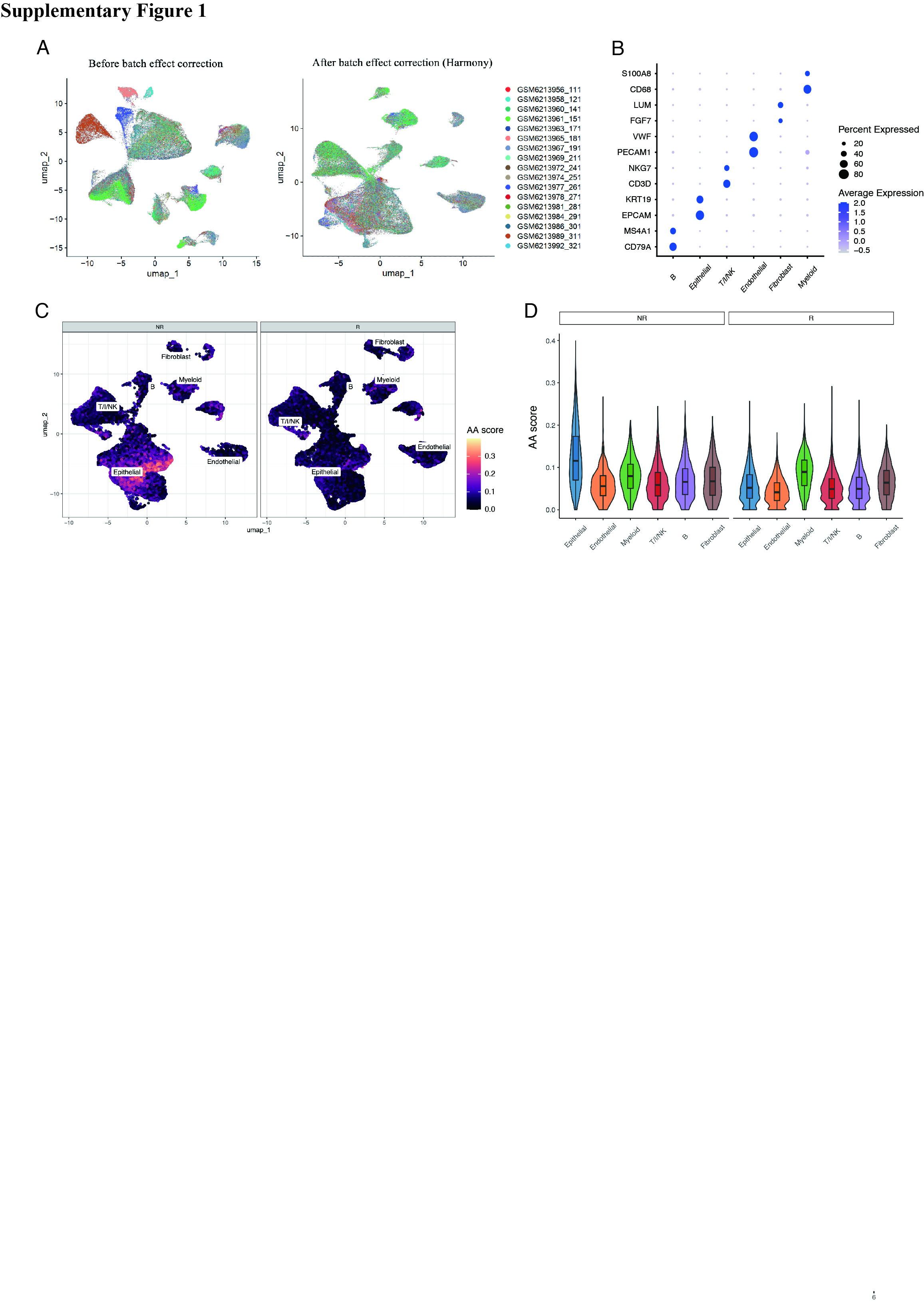

Supplement: Supplementary Figure 1 — Epithelial cell cluster in PD-1 blockade-resistant CRC patients exhibits elevated amino acid metabolism. Related to Figure 1 . (A) UMAP plots of all cells grouped by samples before and after Harmony batch effect correction. (B) Dot plot showing average expression of known markers in indicated cell types. (C) UMAP plots of all cells colored by AA score calculated between NR and R groups. (D) Violin plots presenting each cell type’s AA score across NR and R groups. T/I/NK, T cells/innate lymphocytes/NK. AA score, amino acid score. NR, non-response. R, response. [file Image1.jpeg]

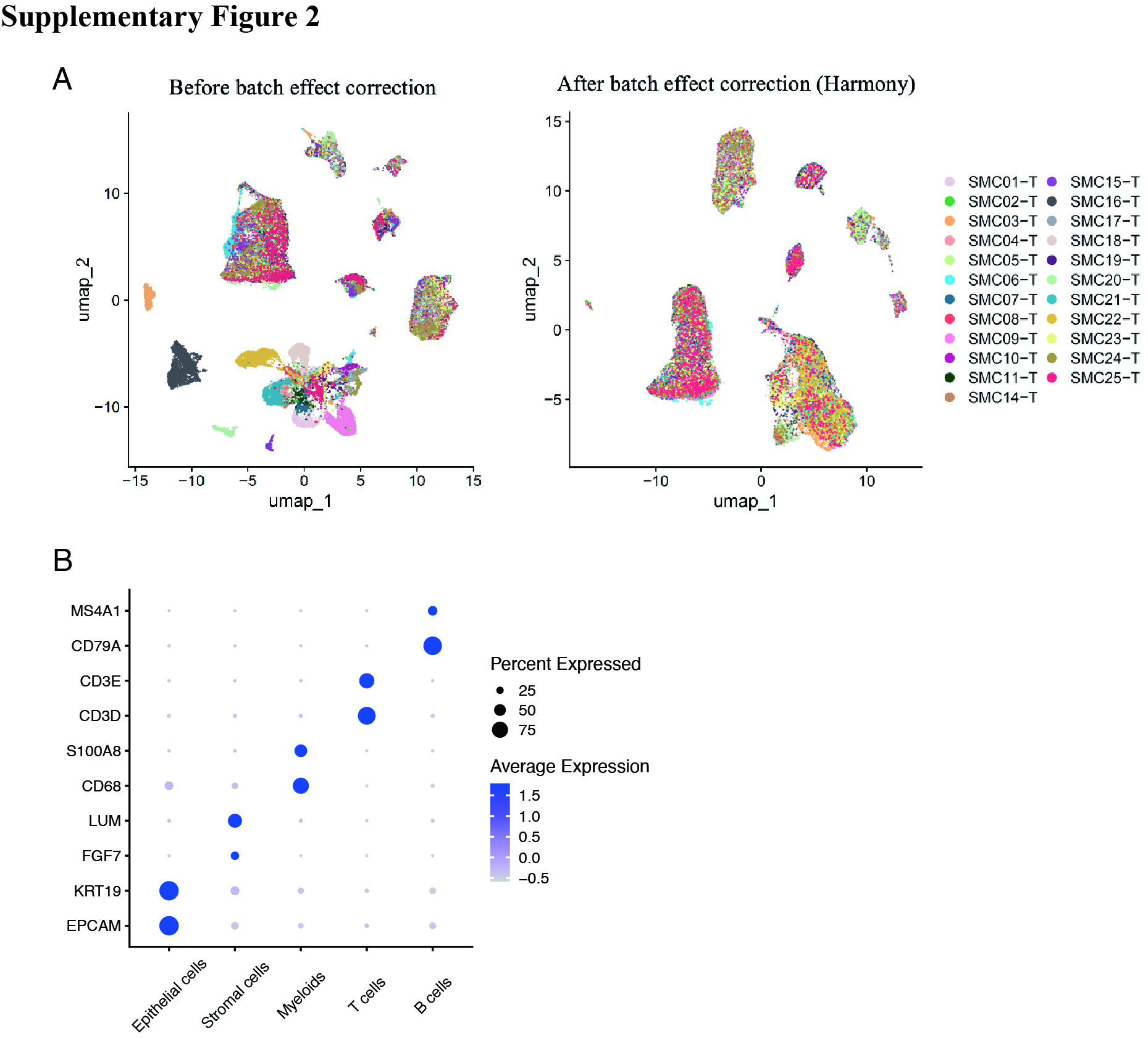

Supplement: Supplementary Figure 2 — Amino acid (AA) score differentiates immune microenvironments in CRC. Related to Figure 2 . (A) UMAP plots of all cells grouped by samples before and after Harmony batch effect correction. (B) Dot plot showing the average expression and percent expression of marker genes across cell types. [file Image2.jpeg]

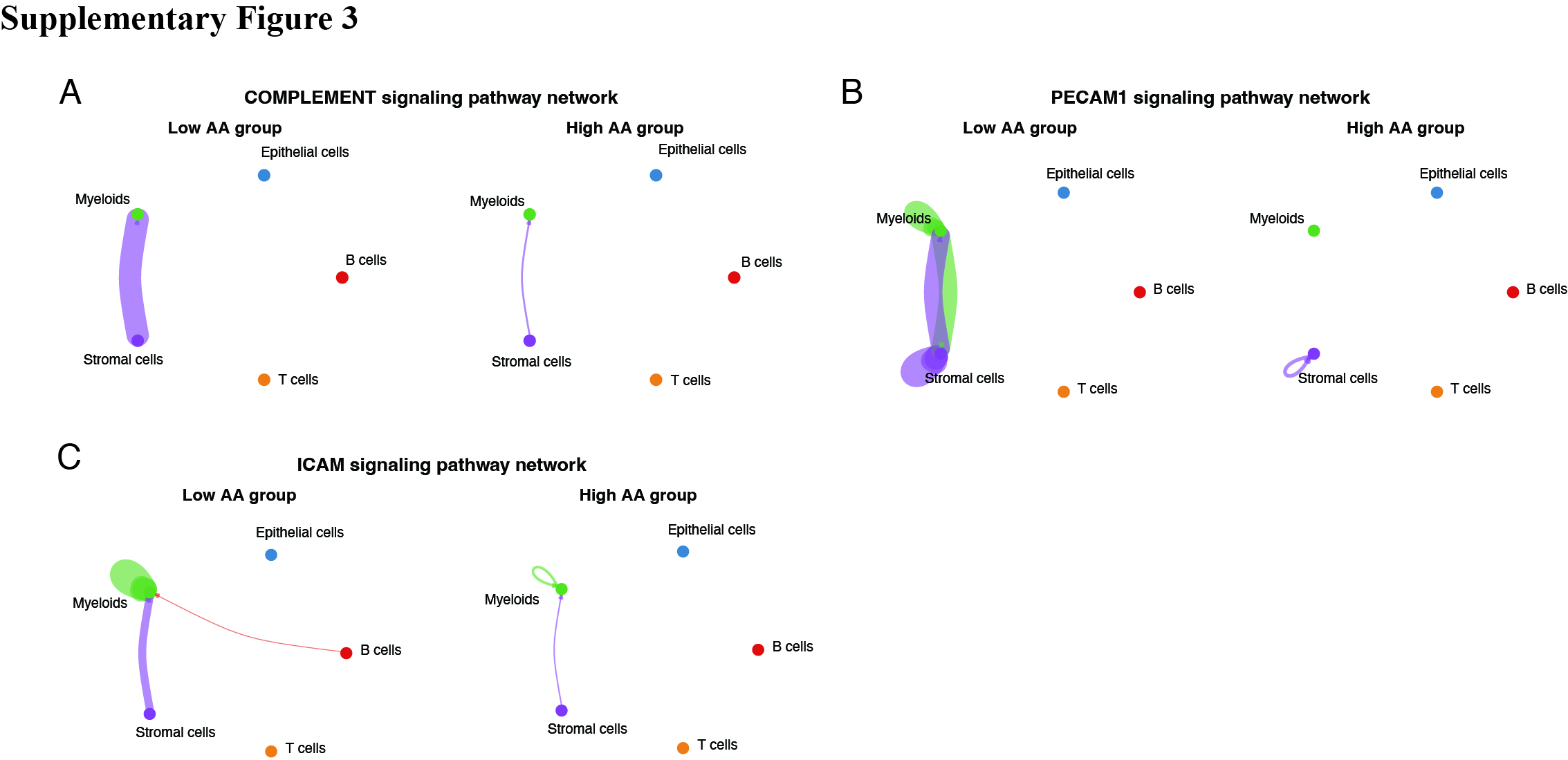

Supplement: Supplementary Figure 3 — Variations in cell-cell interactions and signaling pathways across amino acid (AA) score levels. Related to Figure 3 . (A-C) Circle plots of differences of specific signaling pathways networks between high AA group and low AA group. [file Image3.jpeg]

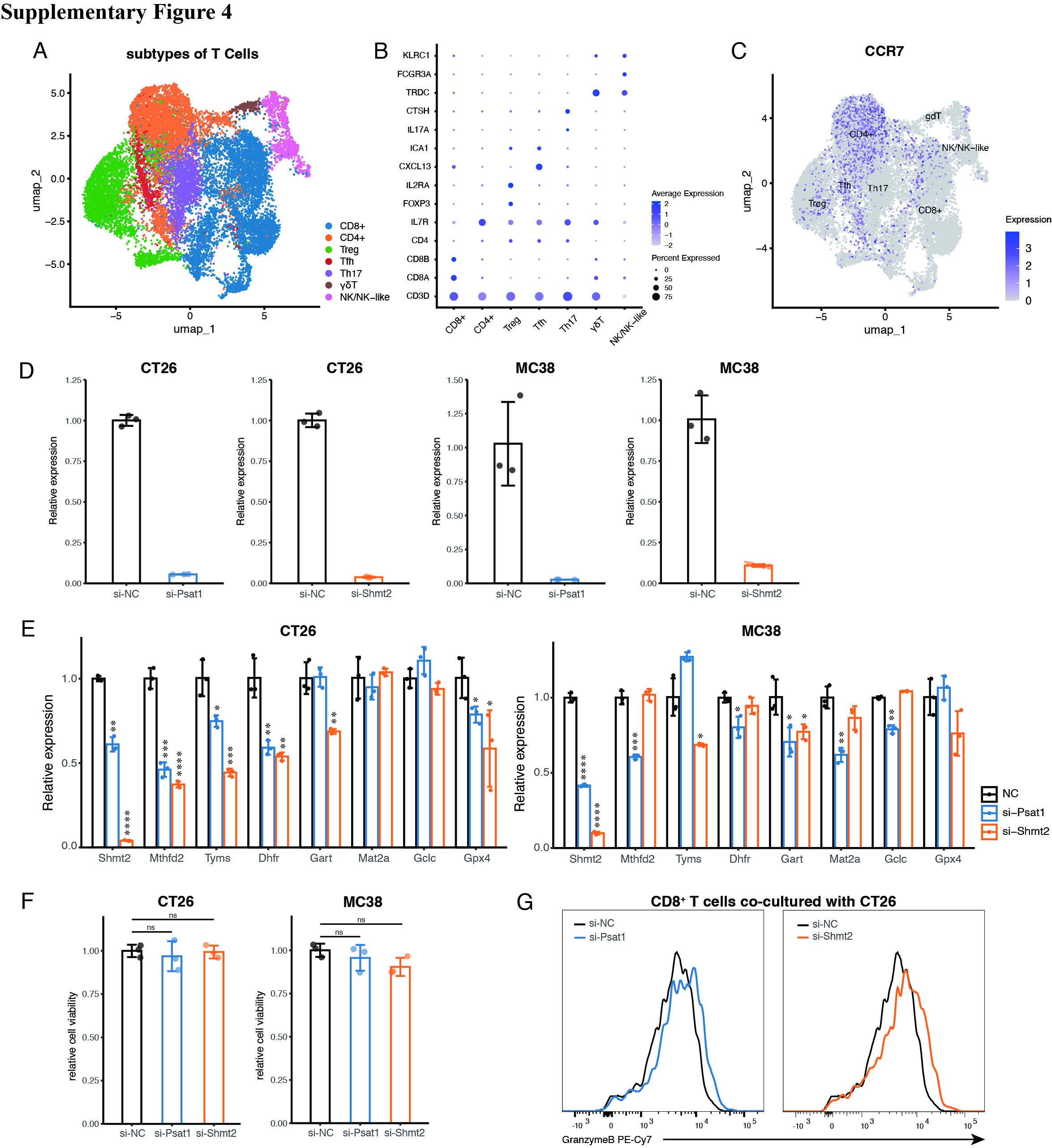

Supplement: Supplementary Figure 4 — Enhanced T cell-mediated immune response in the low amino acid (AA) group. Related to Figure 4 . (A) UMAP visualization of cells from the major T cluster, colored by T cell subsets. (B) Dot plot of representative cell markers among each T subcluster; dot size represents abundance, and color represents expression level. (C) UMAP feature plot of naive marker gene expression of the major T cluster cells. (D) Gene expression levels validated by RT-qPCR after siRNA transfection. (E) qPCR validation of downstream metabolic genes in Psat1- and Shmt2-knockdown CRC cell lines. Significance was assessed by comparing si-Psat1 vs NC and si-Shmt2 vs NC. (F) Histograms of relative cell viability in CT26 and MC38 cells 24 hours post-seeding. (G) Representative histogram of Granzyme B fluorescence intensity in CD8+ T cells co-cultured with CT26 cells. *, P < 0.05; **, P < 0.01; ***, P < 0.001; ns, not significant, P > 0.05. [file Image4.jpeg]

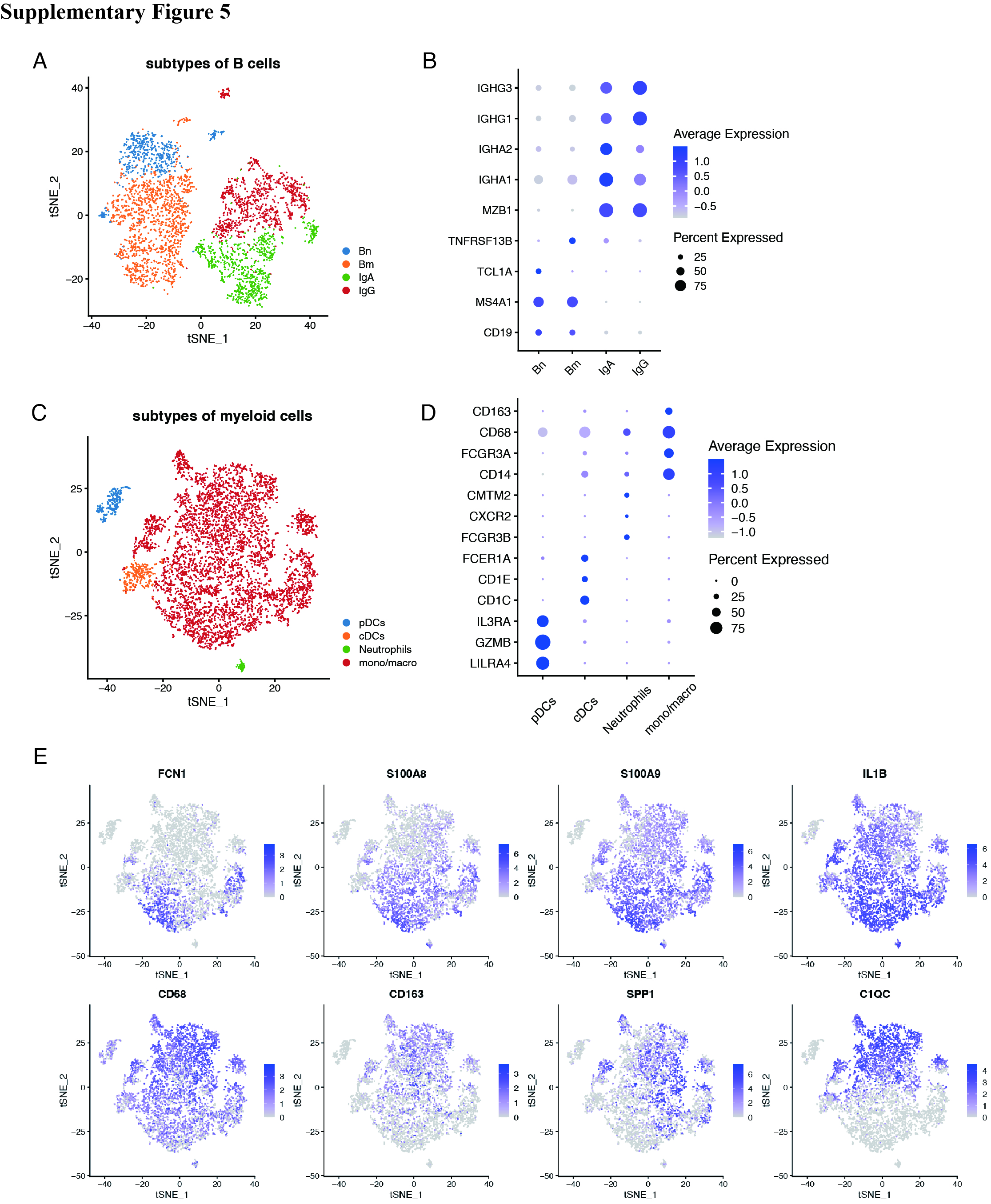

Supplement: Supplementary Figure 5 — Distinct traits of tumor-infiltrating B and myeloid cells between amino acid (AA) groups. Related to Figure 5 . (A) t-SNE visualization of subtypes from major B cell cluster. (B) Dot plot showing the average expression and percent expression of marker genes across B cell subtypes. (C) t-SNE plot of subpopulations from myeloid cluster. (D) Dot plot showing the average expression and percent expression of marker genes across myeloid subtypes. (E) t-SNE feature plots of marker genes of myeloid cells. Bn, naïve B cells. Bm, memory B cells. IgA, IgA+ plasma cells. IgG, IgG+ plasma cells. pDCs, plasmacytoid dendritic cells. cDCs, conventional dendritic cells. mono/macro, monocytes/macrophages. [file Image5.jpeg]

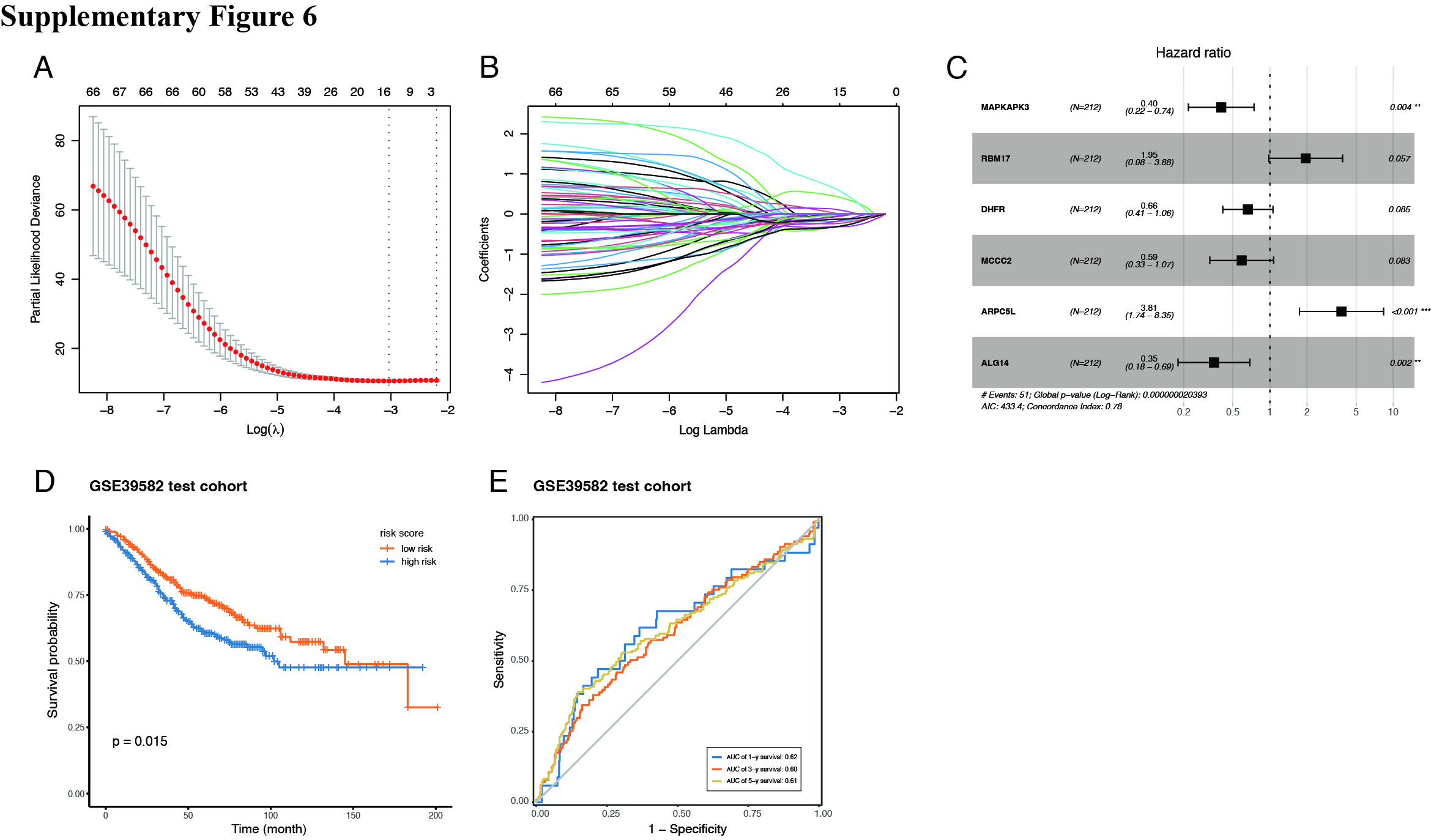

Supplement: Supplementary Figure 6 — Generation and validation of an amino-acid related risk score to reveal prognosis, immune characteristics and therapeutic implications. Related to Figure 6 . (A) LASSO Cox regression analysis of the association between deviance and log(λ). (B) LASSO Cox regression analysis of the association between coefficients of genes and log(λ). (C) Forest plot showing hazard ratio of 6 selected genes. (D, E) The OS discrepancy between the high-risk and low-risk groups and the ROC curve of risk score predicting the 1-, 3-, and 5-year OS using the GSE39582 dataset. [file Image6.jpeg]
